# Predictors of Orthostatic Hypotension in the Elderly: Results from the Amirkola Health and Ageing Project (AHAP) Study 

**Published:** 2019-10

**Authors:** Mehdi Safarpour, Akbar Fotouhi, Seyed Reza Hosseini, Masume Mohamadzade, Ali Bijani

**Affiliations:** 1 *Department of Disease Prevention and Control, Health Deputy, Babol University of Medical Sciences, Babol, Iran.*; 2 *Department of Epidemiology and Biostatistics, School of Public Health, Tehran University of Medical Sciences, Tehran, Iran.*; 3 *Department of Community Medicine, School of Medicine, Babol University of Medical Sciences, Babol, Iran.*; 4 *Children’s Non-Communicable Diseases Research Center, Babol University of Medical Sciences, Babol, Iran.*

**Keywords:** *Hypotension, orthostatic*, *Aged*, *Prevalence*

## Abstract

**Background:** Orthostatic hypotension (OH) in the elderly is an important health challenge that poses a significant burden. We sought to determine the prevalence and correlates of OH in an elderly population-based study.

**Methods:** This study was conducted within the framework of the Amirkola Health and Ageing Project (AHAP) on 1,588 elderly individuals aged ≥60 years. The baseline measurement was performed from April 2011 to July 2012. The relationships between OH (dependent variable) and age, sex, diabetes, hypertension, and cognitive status (independent variables) were investigated by logistic regression.

**Results:** The mean age of the participants was 69.37±7.42 years (men: 69.96±7.68 y, women: 68.66±7.02 y). The prevalence of OH was 10.7%: 8.4% in the male and 13.7% in the female patients. In the final model, hypertension (OR=2.4, 95% CI: 1.6–3.7), diabetes (OR=1.3, 95% CI: 1.0–1.9), age (OR=2.9, 95% CI: 1.7–4.8), and female sex (female [OR=1.6, 95% CI: 1.1–2.3]) were significantly correlated with OH.

**Conclusion: **The prevalence of OH in our elderly subjects increased with age. Additionally, the older participants with diabetes and hypertension had a higher likelihood of having this OH.

## Introduction

According to the definition of the American Academy of Neurology, the criteria for the diagnosis of orthostatic hypotension (OH) are a reduction of up to a minimum of 20 mm Hg in systolic blood pressure or 10 mm Hg of diastolic blood pressure in a person within 3 minutes of standing.^1^ OH may result from a decrease in the amount of intravenous blood, a baroreceptor reaction, and the insufficient response of the sympathetic or cardiovascular system that leads to a reduction in the cardiac output.^2^ OH has been known as one of the symptoms of diabetic autonomic neuropathy.^3^ Elderly people in the aging process are at increased risk of developing neuropathy.^4^ These patients may complain of fatigue, dizziness, mild headaches, and blurred vision. Other uncommon clinical symptoms include pain in the neck and shoulder with concomitant weakness.^5^^, ^^6^ However, in many patients, OH may show no symptoms and can only be detected by accurate measurement of blood pressure in a proper position.^7^

The reports on the prevalence of OH are varied in references, which is probably due to the effect and presence of contributing factors such as the definition of this problem, age, the use of drugs, and associated illnesses in the population under study.^8^^, ^^9^ Joseph et al.^10^ reported the prevalence of OH in the general population to be around 6%, while it increased to 10%–30% with an increase in age, and even up to 50% in some studies in the elderly. A combination of the aging process and diabetes tends to increase the risk of the development and progress of OH in the elderly. Several studies have reported OH prevalence rates of about 28%–30%.^11^^, ^^12^ In this regard, Wu et al.^13^ reported prevalence rates of OH in diabetic patients ranging from 8.2% to 42% and reasoned that this was probably due to the difference in diagnostic criteria and studied populations. Several factors such as age, the duration of diabetes, inappropriate blood glucose control, hypertension, and some diseases cause the development of OH in elderly diabetic patients.^13^^, ^^14^ OH is a risk factor for falls in elderly people.^15^ Further, according to a report, the number of direct OH admissions in the United States was about 80,000 in 2004.^16^ Many fall-prevention programs have been suggested based on the association between OH and falls, although the available evidence regarding this association is contradictory.^11^ Therefore, OH is a significant health challenge and poses a significant burden. Proper management is also a concern for healthcare providers. Unlike hypertension, which is often asymptomatic and requires treatment for the prevention of long-term complications, the management and control of OH is largely dependent on its symptoms and is aimed at improving the patient’s quality of life.^10^ Given the current lack of data on the prevalence of OH in the elderly, we sought to determine this index and its related factors in a population-based study. 

## Methods

The data for the present cross-sectional population-based study were extracted from the Amirkola Health and Ageing Project (AHAP). Amirkola is a small town in northern Iran. The AHAP study is the first comprehensive cohort investigation of the health of older people ever conducted in Iran. The aim of this project was to investigate the health status of the elderly in Amirkola. The participants of the study were comprised of individuals aged ≥60 years who lived in Amirkola. According to the latest census, there were 2,234 people, consisting of 1158 men and 1076 women, aged ≥60 years living in 34 districts in this city. The city inhabitants were informed about the study in talks in mosques and also via posters distributed throughout the city. After the elderly were notified and invited to participate in the study, 1,616 people agreed to be recruited in the research. The interviewers initially visited older people in their homes to complete parts of the study questionnaire and then, on the next day, the participants referred to the Social Determinants of Health Research Centre of the University of Babol in Amirkola to complete questionnaires and examinations and to have fasting blood samples taken for biochemical and hormonal tests. The baseline measurement was performed from April 2011 to July 2012.^17^ The inclusion criteria were age ≥60 years and residence in Amirkola, and the exclusion criterion was unwillingness to participate in the study.

From the 1,616 participants in this study, 28 individuals were excluded due to incomplete information. The study was conducted on 1,588 elderly people aged ≥60 years. The status of OH (dependent variable) was determined via blood pressure measurement using the Omron M3 Intelligence Device in supine and standing positions in 2 steps via a standard method. OH was diagnosed when a fall in systolic blood pressure of at least 20 mm Hg and/or diastolic blood pressure of at least 10 mm Hg within 3 minutes of standing was recorded. Thereafter, the participants were divided into 2 groups of with and without OH. Variables such as age, sex, diabetes, blood pressure, and cognitive status were considered independent variables. Cognitive status was examined using the Mini-Mental State Examination (MMSE). Based on the scores acquired by the participants, they were assigned into normal state as well as mild, moderate, and severe cognitive impairment groups. The presence of diabetes or hypertension in the participants was determined based on both self-report and the participants’ health insurance booklets. 

In data analysis, the quantitative data were described as the mean and the standard deviation together with the median. The qualitative data were described in ratios. The relationship between each of the independent study variables and OH was described using the crude odds ratio (OR). The adjusted OR of the final model was reported using the logistic regression model through a backward approach. The data analyses were conducted using IBM SPSS Statistics for Windows, version 22.0, and 95% confidence intervals were established. The research project of the original study on the health of elderly people in Amirkola was approved during the thirty-second session of the Ethics Committee of the Research Committee of Babol University of Medical Sciences on10/12/2010. Additionally, the thesis project entitled “Investigating the Incidence and Risk Factors and the Predictive Model of Falls in the Elderly of Amirkola: A Population-Based Study” (code: IR.TUMS.VCR.REC.1395.54) was approved by the Ethics Committee of Tehran University of Medical Sciences. 

## Results

According to the results, men accounted for 55.0% of the study population. The mean age of the participants was 69.37±7.42 years (men: 69.96±7.68 y, women: 68.66±7.02 y), the median age was 68 years, and the 25th and 75th percentiles were 63 and 75, respectively. Of the total, 171 (10.7%) participants (95% CI: 9.2–12.3) had OH. The prevalence of OH was higher in the women than in the men and in the age group of 80-year-olds than in the other age groups. Moreover, the prevalence of diabetes was 31.0% (95% CI: 28.7–33.3). The prevalence of OH was higher in the diabetic population (13.8%) than in the other participants (95% CI: 10.8–17.1). According to the results depicted in Table 1, the age group of 60–69-year-olds had the highest frequency and 23.3% of the elderly had varying degrees (mild to severe) of cognitive impairment. In the univariate analysis, the associations between all the independent variables and OH were statistically significant. Nonetheless, in the final model, after the adjustment of the effects of the variables, this association was eliminated for cognitive status. Furthermore, the analysis revealed that the mean systolic blood pressure and the mean diastolic blood pressure in the supine position in the subjects with OH were significantly higher than those in the participants without this problem (P<0.001). 

**Table 1:** Prevalence and odds ratios of orthostatic hypotension by different risk factors

	Frequency n (%)	Number (Prevalence: 95% CI)	Odds ratio: 95% CI	
			Crude	Adjusted
Age Groups			P<0.001	P<0.001
60-69 (y)	950 (59.8)	82 (8.6: 6.9 -10.5)		
70-79 (y)	513 (32.3)	61 (11.8: 9.2-15.0)	1.4: 1.0-2.0	1.3: 0.9-1.9
≥80 (y)	125 (7.9)	28 (22.4: 15.4-30.7)	2.9: 1.8-4.6	2.9: 1.7-4.8
Gender			P<0.001	P=0.003
Male	872 (55.0)	73 (8.4: 6.6-10.4)		
Female	716 (45.0)	98 (13.7: 11.2-16.4)	1.7: 1.2 - 2.3	1.6: 1.1-2.3
Cognitive Status			P<0.001	P=0.518
Normal	1217 (76.6)	114 (9.4: 7.7-11.1)		
Mild disorder	250 (15.7)	40 (16.0: 11.6-21.1)	1.8: 1.2-2.7	1.2: 0.9-1.9
Intermediate and severe disorder	121 (7.7)	17 (14.0: 8.4-21.1)	1.6: 0.9-2.7	2.9: 1.7-4.8
Hypertension			P<0.001	P<0.001
Negative	598 (37.6)	32 (5.3: 3.6-7.4)		
Positive	990 (62.4)	139 (14.0: 11.9-16.3)	2.8: 1.9-4.2	2.4: 1.6- 3.7
Diabetes			P=0.021	P=0.052
Negative	1096 (69.0)	103 (9.3: 7.7-11.2)		
positive	492 (31.0)	68 (13.8: 10.8-17.1)	1.4: 1.1- 2.0	1.3: 1.0-1.9

## Discussion

In the present study, we found a significant correlation between OH and diabetes, hypertension, age, and sex. The prevalence of OH in our study population was 10.7%, and this was 13% in the diabetics. The prevalence of OH is higher in the present study than in other studies that reported a rate of 5.8%–6.9%.^18^^-^^20^ However, this value is equal to the value obtained by Zho et al.,^21^ who reported a rate of 11% in a study conducted in Singapore. In previous studies, the prevalence of OH in diabetic patients is more than twice the rate in the present study (i.e., 25.5%–28%).^11^^, ^^13^^, ^^22^ This contradiction could be justified by the presence of many factors such as age, demographic characteristics, and diabetes which affect the incidence of OH. In addition, the different criteria for diagnosing OH in studies, the underlying diseases such as diabetes, and the duration of the disease can be the other causes of these differences. In confirmation of the above issue and the effect of age, the difference in the prevalence OH can be noted in the 2 age groups of 60–69 and >80 years, as the proportion of the young elderly was about 8%, which grew 2.5 times in elderly parity and reached 22% for 80 years. 

A meta-analysis study reported that there was a relationship between OH and cardiovascular diseases and that OH increased the risk of death by 36%.^23^ Wu et al.^13^ also reported OH as a risk factor for cardiovascular diseases and syncope. Accordingly, many patients with OH may be excluded and the final prevalence does not indicate a real prevalence. In the current study, age had a direct and meaningful relationship with OH, and the prevalence had an increasing trend in both sexes with increasing age. This finding contradicts the results obtained by Budyono et al.,^22^ who did not report the relationship between these 2 variables, while it chimes in with another study.^13^ The lack of association between age and OH can be attributed to premature death.^13^ Physiological mechanisms can justify the association between OH and age because with increasing cardiac compliance,^24^ the weakening of the vestibular sympathetic reflex^25^ and the reduction of the responsiveness of the baroreflex^26^ occur in an individual, and all of these age-related changes may render an individual susceptible to OH. 

Although the mechanism for increasing the prevalence of OH is known in the elderly, the sex difference in the problem is still not well known. In the present study, after adjusting the other variables, we found that our female subjects had a 1.6-fold chance of having OH, which is consistent with the result of another study.^27^ In justifying this phenomenon, it can be said that women are genetically more susceptible to OH than men. The autonomic nervous system of women exhibits more activity in parasympathetic control than that of men.^28^ Put differently, men have a stronger sympathetic system and, hence, have a stronger response to cardiac stress.^29^^, ^^30^ In a postural challenge, women have less ability to compensate hypotension due to the weakness of their sympathetic system.^31^ Along with the differences in the autonomic nervous system between women and men, female hormones play an important role in regulating blood pressure. Female hormones such as estrogen can affect the secretion of norepinephrine, vasopressor, and epinephrine neurotransmitters, which are responsible for increasing the tone of the sympathetic system during orthostasis. Watt et al.^32^ also reported the association between estrogen-induced hypovolemia and vascular estrogen receptors, which can affect the tone of the vessels. The other reason for the difference in OH is the difference in anatomy between the 2 sexes. The gravity center of women is 8%–15% lower than that of men. The gravity center points to the relationship between height and body mass. This ratio in men is taller height to lower mass. By contrast, women have shorter height and more body mass in the lower extremities. Therefore, the gravity center of women with their pelvic proportions is likely to play a role in the birth process. Summers et al.^33^ reported that having a higher mass ratio in the lower extremities of their study participants resulted in blood accumulation in the pelvic environment. Naturally, when blood is collected in the environment, the autonomic nervous system should overcome this problem. With the excessive accumulation of blood in the lower region, the effectiveness of the autonomic nervous system for the correction of this imbalance may be compromised. Simply put, a lower gravity center in women is associated with an increase in blood loss in the lower extremities, resulting in a reduction in blood transfusion to the heart.^34^ Other studies have shown that the difference in the gravity center in women is a direct cause of the low vascular resistance when the position is changed.^35^^, ^^36^

Based on the references, there are 3 hypotheses vis-à-vis the relationship between OH and cognitive impairment. First, neurodegenerative diseases may result in OH and cognitive impairment due to common pathological processes that affect both cognitive space and cardiovascular control. On the other hand, OH may lead to cerebral hypoperfusion, which is likely to contribute to the development of cognitive impairment. Finally, recent data suggest that cognitive impairment should probably be considered a transient sign, rather than a chronic effect.^37^ In other words, some studies have suggested a constant change in blood pressure as a factor with a negative effect on brain perfusion^38^ and introduced blood pressure decrease as a risk factor for cognitive impairment.^39^ In the present study, although there was a relationship between cognitive status and OH in the univariate analysis, this relationship disappeared after we made adjustments for the other variables. The results of this part of our study contradict the results of the studies by Mehrabian et al.^40^ and Bengtsson et al.,^41^ whereas they are consistent with other studies that did not report the relationship between the 2 variables.^42^^-^^44^ The results of the study can be interpreted in several ways. First, the validity of the OH diagnostic test may be a question insofar as the participants were classified into 2 groups of with and without OH based on a single test, which may have reduced the validity of OH diagnosis. Another potential source of bias is the assessment of cognitive abilities. The MMSE is a rigid method for testing cognitive function and focuses more on mental ability than cognitive capacity. The sensitivity of this test is very weak in detecting low cognitive function changes, which can be somehow explained by a lack of communication.^43^ However, the findings of this study should be interpreted with caution because this absence of communication may be due to other causes and variables that are not included in this study. 

In the current study, the subjects with a history of hypertension had a 2.4 higher likelihood of having OH than their counterparts without OH. The association between hypertension and OH has also been reported in other studies.^13^^, ^^18^^, ^^45^ In the interpretation of the above result, it can be argued that individuals with hypertension are likely to consume medications that can lower blood pressure and, on the other hand, reduce the cardiac output and blood volume. In addition, research suggests that high blood pressure is generally associated with a reduction in cardio acceleration, baroreflex-mediated responses, vasoconstriction, renal salt and water conservation, and slow cardiac filling, thereby increasing the OH risk in the elderly.^46^ Other reasons include the fact that individuals with OH have a high mean systolic and diastolic blood pressure in sleeping status compared with subjects without OH. Higher average blood pressure in the supine position may be allied to a drop in blood pressure in the standing position. Vessel dysfunction is likely to cause carotid artery and aortic failure to maintain orthostatic stress in the bloodstream,^12^ which was the case in our study, where we found that the mean value of blood pressure (systolic and diastolic) in resting conditions was higher in the patients with OH than in the subjects without OH.

In the present study, the subjects with diabetes had a 30% higher chance of having OH than the subjects without a history of diabetes, and the association between diabetes and OH was statistically significant. This finding is in line with other studies that reported a relationship between OH and a history of diabetes.^11^^, ^^13^^, ^^22^ Purewal et al.^2^ cited other studies reporting that OH might be a combination of several factors such as an abnormal reduction in the amount of plasma in the standing position; a reduced cardiac output associated with a reduced vascular return, the inability to increase heart rate, or a weakened contraction of the heart; and impaired peripheral vasoconstriction.

In diabetic neuropathy, all of the above mechanisms can be effective. Neuropathy in diabetes affects both vasomotor and cardiac nerve systems. The evidence from the literature suggests that impaired peripheral vasoconstriction is deemed the main deficiency in OH caused by diabetic neuropathy. Tsutsu et al.^47^ reported that their subjects with OH had a history of diabetes for 10 years or more, while their subjects without OH had diabetes for usually less than 5 years. Previous studies have provided evidence of an association between the continuation of hyperglycemia and the development and progression of diabetic neuropathy, which can be explained by the question “Why do people with OH have a tendency to a longer period of diabetes?”.^48^

The population-based nature of the present study and its appropriate sample volume should be regarded as its strong points. Nevertheless, that we did not consider the drugs consumed by our elderly patient population to be a potential predictor of OH is one of the weaknesses of our study. 

## Conclusion

The results of the current study underscore the issue of orthostatic hypotension in the elderly, indicating that its prevalence is in tandem with an increase in age. Additionally, our older subjects with chronic underlying conditions such as diabetes and hypertension had a higher likelihood of having orthostatic hypotension. We, therefore, suggest that orthostatic hypotension be granted further consideration by healthcare providers in the evaluation of older people with underlying illnesses.

## Notes:

***This paper should be cited as: ***Safarpour M, Fotouhi A, Hosseini SR, Mohamadzade M, Bijani A. Predictors of Orthostatic Hypotension in the Elderly: Results from the Amirkola Health and Ageing Project (AHAP) Study. J Teh Univ Heart Ctr 2019;14(4): 165-170.
